# SIRT1-dependent myoprotective effects of resveratrol on muscle injury induced by compression

**DOI:** 10.3389/fphys.2015.00293

**Published:** 2015-10-21

**Authors:** Thomas K. Sin, Benjamin Y. Yung, Shea P. Yip, Lawrence W. Chan, Cesar S. Wong, Eric W. Tam, Parco M. Siu

**Affiliations:** ^1^Department of Health Technology and Informatics, Faculty of Health and Social Sciences, The Hong Kong Polytechnic UniversityHong Kong, Hong Kong; ^2^Interdisciplinary Division of Biomedical Engineering, Faculty of Engineering, The Hong Kong Polytechnic UniversityHong Kong, Hong Kong

**Keywords:** compression injury, pressure ulcer, resveratrol, SIRT1, skeletal muscle

## Abstract

Our current understanding on the molecular mechanisms by which sustained compression induces skeletal muscle injury is very limited. This study aimed to test the hypothesis that activation of SIRT1 by the natural antioxidant resveratrol could deactivate apoptotic and catabolic signaling in skeletal muscle exposed to moderate compression. Two cycles of 6-h constant pressure at 100 mmHg was applied to the tibialis region of right, but not left hindlimbs of Sprague Dawley rats pre-treated with DMSO (vehicle control) or resveratrol with/without sirtinol. Skeletal muscle tissues lying underneath and spatially corresponding to the compressed sites were collected for analyses. Resveratrol prevented the compression-induced manifestations of pathohistological damages including elevations of the number of interstitial nuclei and area of interstitial space and ameliorated oxidative damages measured as 4-hydroxy-2-nonenal (4HNE) and nitrotyrosine in skeletal muscle. In parallel, resveratrol augmented the expression level and activity of SIRT1 and phosphorylation levels of Foxo3a and Akt while suppressed the increases in protein abundances of p53, Bax, MAFbx, and ubiquitin, enzymatic activities of caspase 3 and 20S proteasome, and apoptotic DNA fragmentation in the compressed muscle. These favorable myoprotective effects of resveratrol were diminished upon pharmacological blockade of SIRT1 by using sirtinol. These novel data support the hypothesis that the anti-apoptotic and anti-catabolic effects of resveratrol on compression injury in skeletal muscle required the action of SIRT1.

## Introduction

Pressure ulcer (PU) refers to tissue ulceration at epidermal and/or sub-dermal levels in response to sustained compression by supporting interfaces such as beds, wheelchairs and prosthetics. It has been unraveled in a retrospective analysis that PU was diagnosed in 4.5% of patients aged between 75 and 85 years upon admission in which 16.7% of these patients developed new ulcers during hospitalization; leading to a ~2.4-fold increase in length of hospital stay (Moore, 2013). Screening of high-risk patients is also difficult; provided that late-stage ulcers often are not diagnosed until open wound beds and concomitant exposure of tendons, skeletal muscle, and/or bones become evident (EPUAP, 2009). Although gross factors including friction and shear were correlated highly with abnormal heel scans, the efficacy of diagnosis was often challenged by the presence of skin alterations (Helvig and Nichols, 2012). Taken together the fact that current interventions for chronic venous leg ulcers depend largely on wound management whereas a considerable number of those are only palliative (Lazarus et al., 2014), understanding the molecular mechanisms of PU is of high importance in drug target identification and design of therapeutics.

The concept of “Deep Tissue Injury” was introduced by the National Pressure Ulcer Advisory Panel (NPUAP) to recognize the form of PU that develops as a consequence of damages of the underlying soft tissues (Stausberg and Kiefer, 2009). Earlier experiments have identified a modest drop in temperature in the compressed skeletal muscle secondary to the occlusion of microvasculature (Linder-Ganz and Gefen, 2007). However, it was proposed that the effectiveness of thermal monitoring could have been precluded by existing variations of body composition among patients and concurrent heat release in surrounding regions with inflammation (Gefen, 2009). While the pathogenic mechanisms of deep tissue injury is largely unknown, a pioneer study has reported that compression stress induced substantial elevations of cell death and stiffness in the skeletal muscle in a dose-dependent fashion (Gefen et al., 2005). Corroborating data from our laboratory have indicated that apoptosis was activated in the skeletal muscle of rats exposed to sustained, moderate compression. Importantly, the increases in apoptotic markers measured as TUNEL index, apoptotic DNA fragmentation and cleavage of caspase 3 in the compressed muscle were observed in circumvent with the presence of pathohistological damages; suggesting that apoptosis might mediate the pathogenesis of deep tissue injury (Siu et al., 2009). This speculation was supported by the data showing that the aforementioned changes were blunted significantly in response to the administration of z-VAD-fmk, which is a caspase inhibitor (Teng et al., 2011).

The thought that oxidative stress is a stimulus of apoptotic cell death prompted us to study the role of oxidative damages in compression-induced injury in the skeletal muscle. We have demonstrated recently that the immunoreactivities of 4HNE, nitrotyrosine and 8-OHdG, which are markers indicative of lipid peroxidation, protein nitrosylation and oxidation of guanine nucleotides respectively, were increased remarkably in the compressed muscle (Sin et al., 2013). In contrast, these elevations were alleviated significantly in animals pre-treated with resveratrol, which is a natural antioxidant commonly found in grapes and berries (Sin et al., 2013). Sirtuin 1 (SIRT1) is the mammalian ortholog of *sir2* in yeasts, in which its activation by resveratrol was accompanied by increased genomic stability and lifespan extension (Howitz et al., 2003). Another line of evidence has suggested that resveratrol prevented the elevations of muscle atrophic factors including MAFbx and MuRF-1 in cultured myotubes under catabolic challenge with dexamethasone (Alamdari et al., 2012). Intriguingly, transcriptional silencing of SIRT1 was found to abolish the protective effects of resveratrol in dexamethasone-treated myotubes (Alamdari et al., 2012). Hence, this study aimed to investigate whether resveratrol would suppress the apoptotic/catabolic machinery in skeletal muscle through a SIRT1-dependent mechanism in response to the induction of compression injury.

## Materials and methods

### Induction of compression injury in the skeletal muscle

Male Sprague-Dawley rats (*n* = 4 per group) were subject to a compression protocol that was established by our laboratory (Sin et al., 2013). Animals were first anesthetized with a mixture of ketamine and xylazine prior to the shaving of hairs of the right hindlimbs. A compression indentor was used to apply a constant pressure of 100 mmHg to the tibialis region of the right hindlimb for 6 h by which the compression force was monitored by a three-axial transducer. This was followed by 18 h of rest whereas the left hindlimb was uncompressed serving as intra-animal control. Complete anesthesia was maintained during the entire compression period through the delivery of 1/3 the anesthetic mixture when deemed necessary. The rats were then exposed to the same protocol the following day such that two cycles of 6-h compression were achieved before sacrifice for the collection of skeletal muscle. All procedures of animal husbandry and compression were conducted in full accordance with the approval of the Animal Subjects Ethics Subcommittee of The Hong Kong Polytechnic University.

### Administration of drugs of interest

The animals were randomly assigned to receive one of the following treatments: DMSO vehicle, resveratrol or a combination of resveratrol and sirtinol (a SIRT1 inhibitor) of either dose at 2 or 5 mg kg^−1^ day^−1^. Resveratrol (RSV) was injected intraperitoneally at a dose of 25 mg kg^−1^ day^−1^ (Sin et al., 2013) in both resveratrol and resveratrol and sirtinol combination groups. In the combination treatment group, 2 or 5 mg kg^−1^ day^−1^ sirtinol (Sin et al., 2015a) was administered immediately through the intraperitoneal route after resveratrol injection. The corresponding amount of DMSO vehicle was administered likewise in the DMSO group. Compression was initiated at once when all the drugs were delivered.

### Haematoxylin and eosin staining

Ten micrometer thick cross sections of skeletal muscle lying underneath the skin of compressed regions were prepared in a freezing cryostat at −20°C. Air-dried sections were then fixed with 10% formalin (HT-5011, Sigma-Aldrich, St Louis, MO, USA) at room temperature for 10 min followed by counter-staining in Mayer's haematoxylin solution (MHS-1, Sigma-Aldrich) for 45 min and 1% eosin in CaCl_2_ (31,8906, Sigma-Aldrich) for 1 min. Numbers of interstitial nuclei and muscle nuclei and area of interstitial space were quantified through the use of Image J software of National Institutes of Health. All the histological data were presented as average results from three random, non-overlapping image fields captured under a 20x objective.

### Immunoblotting

Cytoplasmic and nuclear proteins were extracted from muscle homogenates as described (Sin et al., 2013). Thirty micrograms of proteins were loaded and separated on 10% polyacrylamide gels followed by transfer to PVDF membranes (Immobilon P, Millipore, Billerica, MA, USA). The membranes were blocked for 1 h at room temperature followed by an overnight incubation with the following primary antibodies at 4°C: anti-SIRT1 (15,404, Santa Cruz, Santa Cruz, CA, USA), anti-nitrotyrosine (32,731, Santa Cruz), anti-4-hydroxy-2-nonenol or anti-4HNE (24,327, Oxis), anti-p-Foxo3 (9466, Cell Signaling, Beverly, MA, USA), anti-Foxo3 (97-702, Millipore), anti-p-Akt (9271, Cell Signaling), anti-Akt (9272, Cell Signaling), anti-p53 (56,179, Santa Cruz), anti-Bax (493, Santa Cruz), anti-Bim (11,425, Santa Cruz), anti- MAFbx (33,782, Santa Cruz), anti-MuRF-1 (32,920, Santa Cruz), and anti-ubiquitin (3936, Cell Signaling). The membranes were then incubated with the corresponding secondary antibodies for 1 h. After washing, chemiluminscent signal was then detected using a Kodak 4000R Pro camera with the application of luminal reagent (NEL103001EA, Perkin Elmer, Waltham, MA, USA). All protein expression data were normalized to the signal of β-tubulin (T0198, Sigma-Aldrich) except that of nuclear Foxo3a; of which normalization to histone 2b (07-371, Upstate, Charlottesville, VA, USA) was performed.

### Real time PCR analyses

RNA extraction, reverse transcription and real time PCR were performed according to our previous publication (Sin et al., 2013). The following forward and reverse primers were used: SIRT1: F 5′-TTTCAGAACCACCAAAGCG-3′, R 5′-TCCCACAGGAAACAGAAACC-3′; Nampt: F 5′-GATTCTGGAAATCCGCTCGA-3′, R 5′-TGACTCTAAGGTAAGGTGGCAGC-3′; PSMA2: F 5′-TGGGTCCAGATTACAGAGTC-3′, R 5′-ATGGACGAACACCACCTGA-3′; PSMA5: F 5′-GGACCTTCGTACAGTGTGAT-3′, R 5′-GCTTCTCCTCCATGACTTGC-3′ and PSMB4: F 5′-GTGTAGCTTATGAAGCCCCTTCA-3′, R 5′-ACTCAGCACCGGTTGCTTCT-3′. All data of gene expression were presented as relative fold changes to that of β-tubulin: F 5′-CCTGCTCATCAGCAAGATTCG-3′, R 5′-GTGGTGAGCTTAAGGGTACGG-3′.

### SIRT1 deacetylation assay

The deacetylase activity of SIRT1 was assessed by a fluorometric kit in accordance with the instructions of manufacturer (Cyclex, Nagoya, Japan). Each reaction was initiated by the addition of 5 μl protease inhibitor-free muscle protein extracts under thorough mixing. Fluorescence intensity was measured by a microplate fluorometer for every 2-min time interval. The data were expressed as the rate of reaction per 1 mg of proteins.

### Caspase 3 activity assay

Protease activity of caspase 3 activity was assessed by a fluorometric approach involving the use of caspase 3 substrate DEVD-AFC (1007-200, Biovision, Milpitas, CA, USA). All procedures were adhered to those as described (Teng et al., 2011).

### Cell death ELISA assay

Cell death ELISA assay (Roche Diagnostics, Indianapolis, IN, USA) was conducted to determine apoptotic DNA fragmentation according to the manufacturer's recommendations.

### Proteasome activity assay

The activity of proteasome activity was measured based on the release of fluorescent AMC from its tagged peptide substrate in the presence of proteolytic activity. All procedures were carried out under the recommendations of the manufacturer (K245-100, Biovision).

### Statistical analyses

Statistical analyses were conducted using the SPSS 22.0 software package (IBM, Chicago, IL, USA). A normality test was performed to examine data distributions. All data were expressed as means ± standard error of the mean (SEM). Comparisons were made by One-way ANOVA followed by Tukey *post-hoc* test. Statistical significance was considered at *p* < 0.05.

## Results

### Resveratrol attenuated compression injury through SIRT1

**Figure 1:**
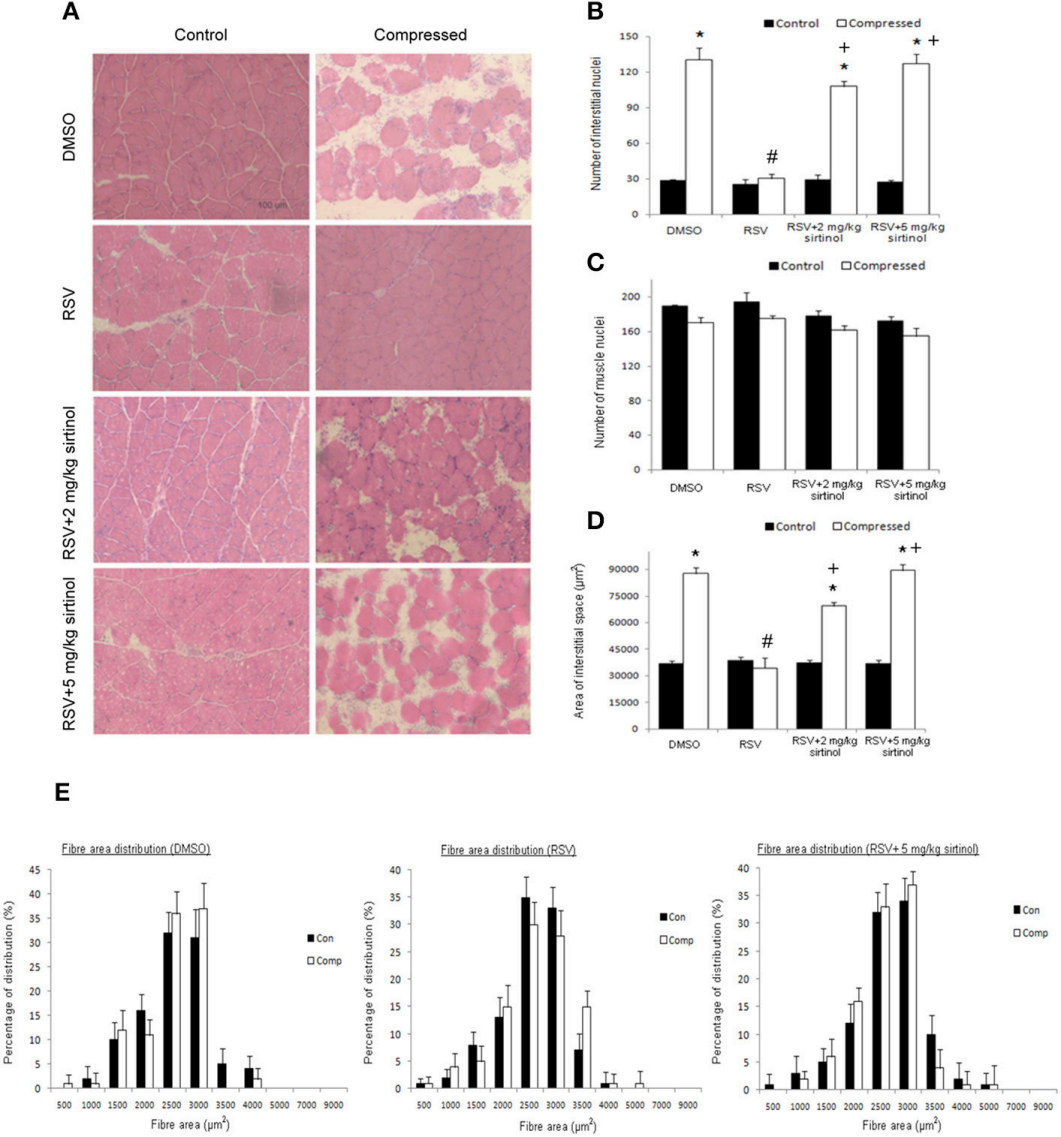
**Histological analyses**. Resveratrol abolished aberrant muscle morphology induced by compression but this effect was reversed by sirtinol **(A)**. The reduction of number of interstitial nuclei by resveratrol in the compressed muscle was mitigated by sirtinol **(B)**. No compression or treatment effects were observed for the number of muscle nuclei **(C)**. The elevation of area of interstitial space induced by compression was alleviated only by resveratrol alone **(D)**. Neither did compression nor drug treatments affect the cross-sectional area of muscle fibers **(E)**. ^*^p < 0.05, compressed muscles compared to uncompressed control muscles; ^#^*p* < 0.05, resveratrol relative to DMSO; ^+^*p* < 0.05, resveratrol + sirtinol compared with resveratrol.

Representative micrographs revealing the effects of moderate compression and treatment of interest on muscle histology are shown (Figure 1A). Our histological analyses indicated that the number of interstitial nuclei was 4.6 folds higher in the compressed muscle relative to the control muscle of DMSO vehicle group (Figure 1B). This compression-induced increase was suppressed by resveratrol, but not in combination with both doses (2 and 5 mg kg^−1^) of sirtinol (Figure 1B). However, the number of muscle nuclei between control and compressed muscles did not differ significantly among all the treatment groups (Figure 1C). In addition, the area of interstitial space in compressed muscles was increased by 2.4 folds compared with control muscles in DMSO group (Figure 1D). Resveratrol, but not in conjunction with sirtinol of either dose, hampered the elevation of area of interstitial space in muscles exposed to moderate compression (Figure 1D). Based on the observation that the inhibitory effects of sirtinol (i.e., increases in number of interstitial nuclei and area of interstitial space in compressed muscles) were more prominent with higher doses (Figures 1B,D), muscle samples arose from 5 mg kg^−1^ sirtinol treatment were then used for subsequent analyses. Furthermore, the distribution of cross sectional area of muscle fibers did not change after compression irrespective of the drugs studied (Figure 1E).

### Inverse regulation of SIRT1 and oxidative stress in the compressed muscle

**Figure 2:**
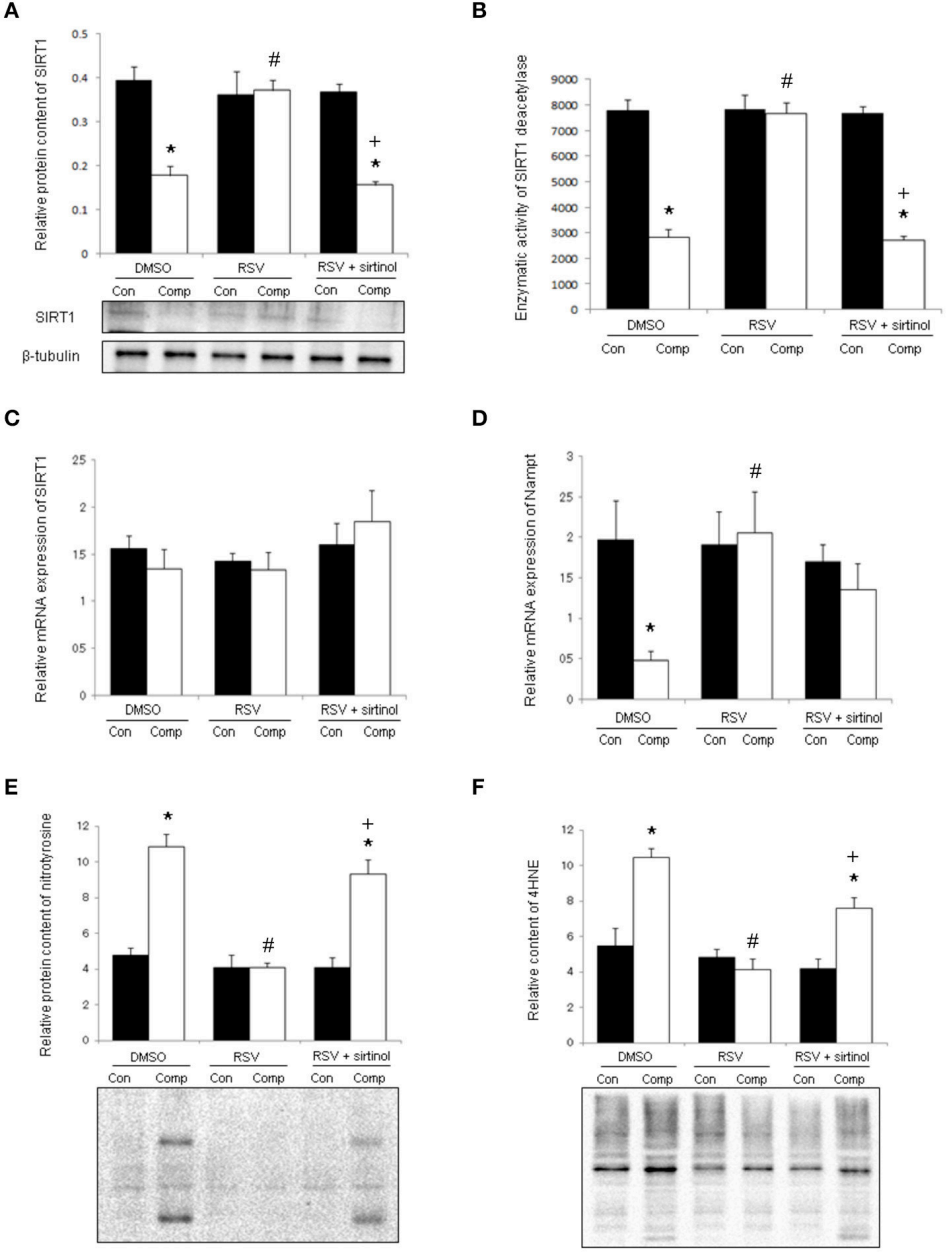
**SIRT1 and oxidative damages**. Resveratrol, but not in conjunction with sirtinol (5 mg kg^−1^), blunted the decrease in protein level of SIRT1 in compressed muscles **(A)**. Similar observations were seen with the deacetylase activity of SIRT1 **(B)**. The mRNA content of SIRT1 **(C)**, but not Nampt **(D)**, was unaffected by compression. The compression-induced increases in nitrotyrosine **(E)** and 4HNE **(F)** were attenuated by resveratrol only in the absence of SIRT1 inhibition. ^*^p < 0.05, compressed muscles (comp) compared to uncompressed control muscles (con); ^#^*p* < 0.05, resveratrol relative to DMSO; ^+^*p* < 0.05, resveratrol + sirtinol compared with resveratrol.

In DMSO vehicle-treated animals, the protein content of SIRT1 was reduced significantly by 55% in the skeletal muscle in response to compression (Figure 2A). This compression-induced reduction was abolished by resveratrol, but not in conjunction with sirtinol (Figure 2A). The reduction of SIRT1 protein in compressed muscles of DMSO-treated rats was followed by a 64% attenuation of SIRT1 deacetylase activity (Figure 2B). This reduction was blunted by resveratrol only in the absence of sirtinol (Figure 2B). The transcript level of SIRT1 between compressed and uncompressed muscles, however, did not differ significantly in all treatment groups (Figure 2C). The mRNA content of Nampt was reduced significantly by 76% in response to moderate compression; this down-regulation was reversed by resveratrol regardless of administration with sirtinol (Figure 2D). Nitrotyrosine and 4HNE are markers of oxidative stress indicative of protein nitrosylation and lipid peroxidation, respectively. The levels of nitrotyrosine and 4HNE were 2.2 folds and 1.9 folds higher in the compressed muscle compared with control muscle in the DMSO group whereas these elevations were not observed in the resveratrol group (Figures 2E,F). The reductions of nitrotyrosine and 4HNE induced by resveratrol, however, were reversed upon SIRT1 inhibition (Figures 2E,F).

### Resveratrol inhibited aberrant phosphorylation status of Foxo3a/Akt

**Figure 3:**
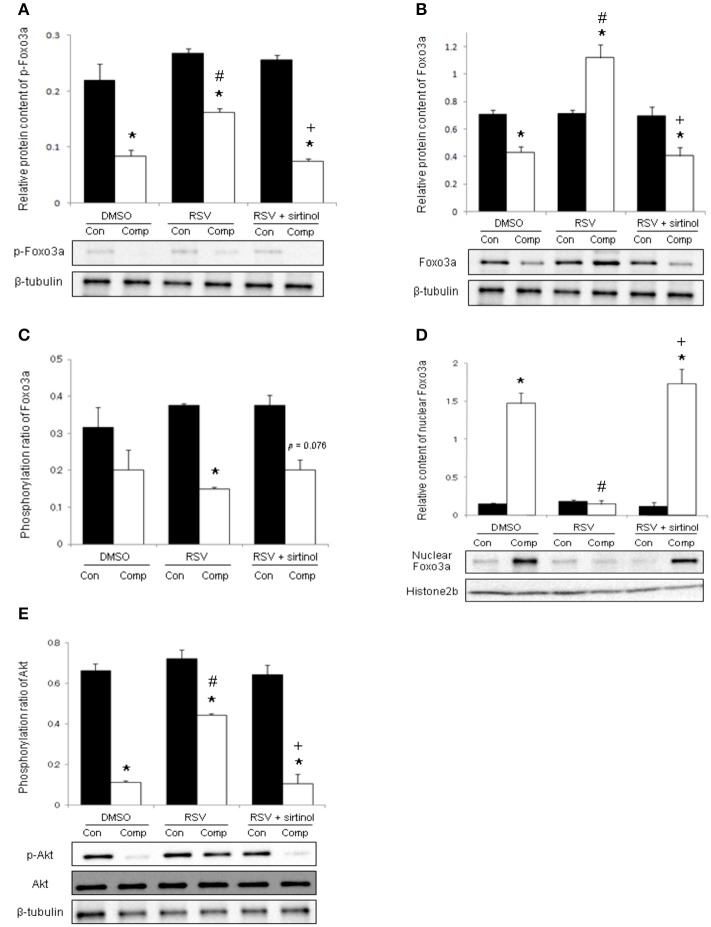
**Phosphorylations of Foxo3a and Akt**. The decrease in phosphorylated Foxo3a in compressed muscle was blunted by resveratrol, but not in combination with sirtinol (5 mg kg^−1^) **(A)**. Resveratrol augmented the cytoplasmic level of Foxo3a **(B)** while suppressed phosphorylation ratio of Foxo3a **(C)** and nuclear Foxo3a **(D)** in compressed muscles. The attenuation of phospho-Akt induced by compression was hampered by resveratrol in the absence of sirtinol **(E)**. ^*^p < 0.05, compressed muscles (comp) compared to uncompressed control muscles (con); ^#^*p* < 0.05, resveratrol relative to DMSO; ^+^*p* < 0.05, resveratrol + sirtinol compared with resveratrol.

The phosphorylation level of Foxo3a was reduced significantly by 62% in the skeletal muscle after compression whereas this reduction was ameliorated by resveratrol administration (Figure 3A). The cytoplasmic protein content of Foxo3a was decreased by 39% in compressed muscles of vehicle-treated rats but not in their counterparts treated with resveratrol (Figure 3B). The phosphorylation ratio of Foxo3a in the vehicle group remained unchanged after compression whereas this ratio was decreased by 60% in resveratrol-treated rats (Figure 3C). In contrast, the nuclear content of Foxo3a was 9.6 holds higher in compressed muscles compared with control muscles in the vehicle group whereas this elevation was abolished by resveratrol (Figure 3D). Of note, these resveratrol-induced alterations were antagonized by combination treatment with sirtinol (Figures 3A,B,D). Moderate compression diminished the phosphorylation level, but not the total protein abundance of Akt by 83% (Figure 3E). This compression-induced reduction, however, was mitigated by resveratrol only in the absence of sirtinol (Figure 3E).

### SIRT1 mediated the anti-apoptotic effects of resveratrol

**Figure 4:**
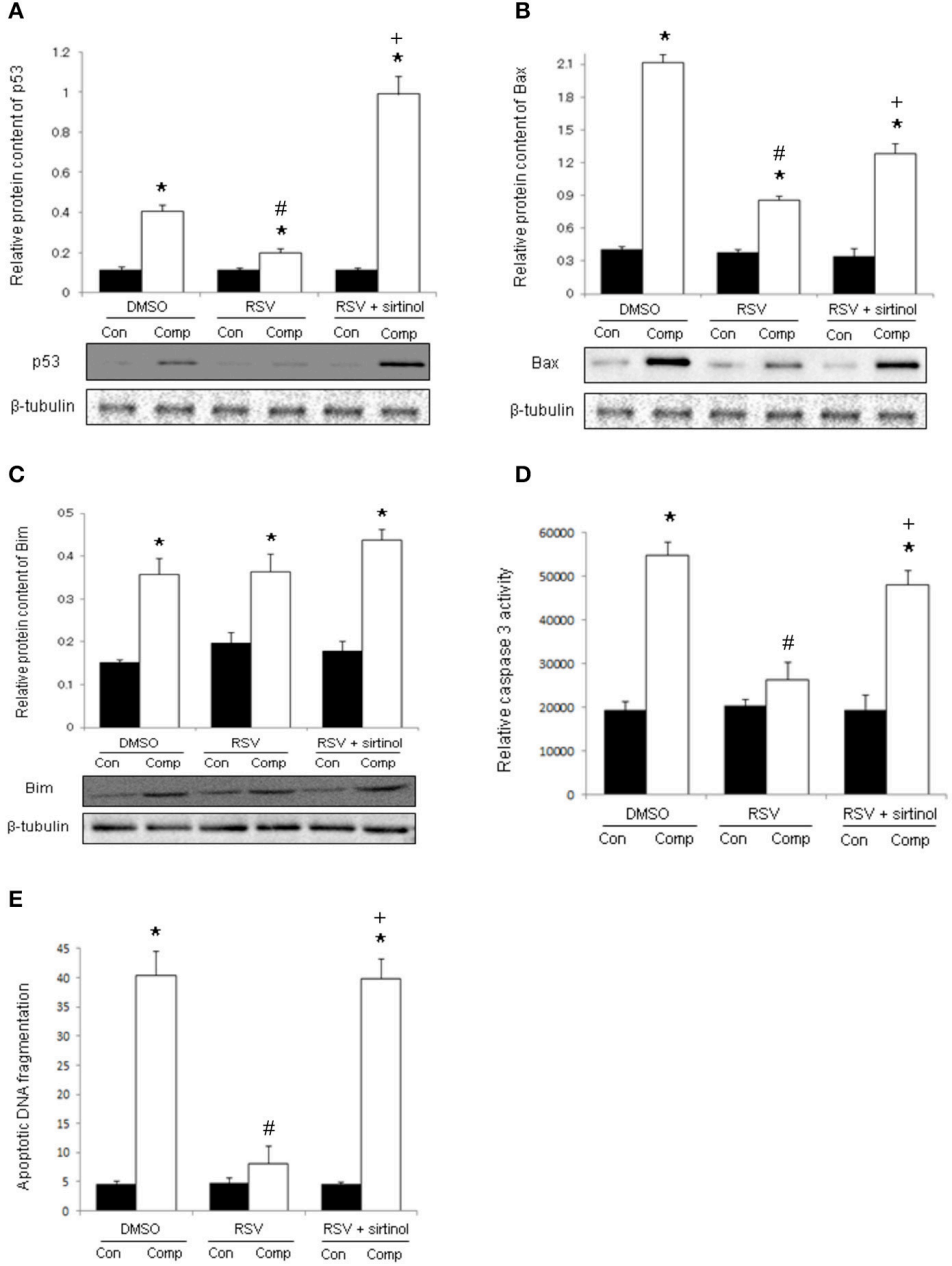
**Signal transduction of apoptosis**. Our immunoblot analyses revealed the suppressive effects of resveratrol, but not in conjunction with sirtinol (5 mg kg^−1^), on the expression of p53 **(A)** and Bax **(B)** in compressed muscles. The up-regulation of Bim by moderate compression was unaffected by any of the drug studied **(C)**. The anti-apoptotic effects of resveratrol were confirmed by the determination of caspase 3 activity **(D)** and apoptotic DNA fragmentation **(E)**. ^*^p < 0.05, compressed muscles (comp) compared to uncompressed control muscles (con); ^#^*p* < 0.05, resveratrol relative to DMSO; ^+^*p* < 0.05, resveratrol + sirtinol compared with resveratrol.

In the DMSO vehicle group, the protein abundances of p53 and Bax were remarkably higher in the compressed muscle relative to control muscle by 3.7 folds and 5.2 folds, respectively (Figures 4A,B). These up-regulations were prevented by resveratrol, but not in combination with sirtinol (Figures 4A,B). The protein expression of Bim was elevated remarkably by 5.3 folds in the compressed muscle but this increase was not affected by any of the treatments (Figure 4C). Compared with control muscles, caspase 3 activity and apoptotic DNA fragmentation were 2.8 folds and 9.0 folds higher respectively in compressed muscles (Figures 4D,E). Resveratrol, but not in combination with sirtinol, reversed the increases in caspase 3 activity and apoptotic DNA fragmentation induced by compression injury (Figures 4D,E).

### Suppression of catabolic markers by resveratrol requires SIRT1

**Figure 5:**
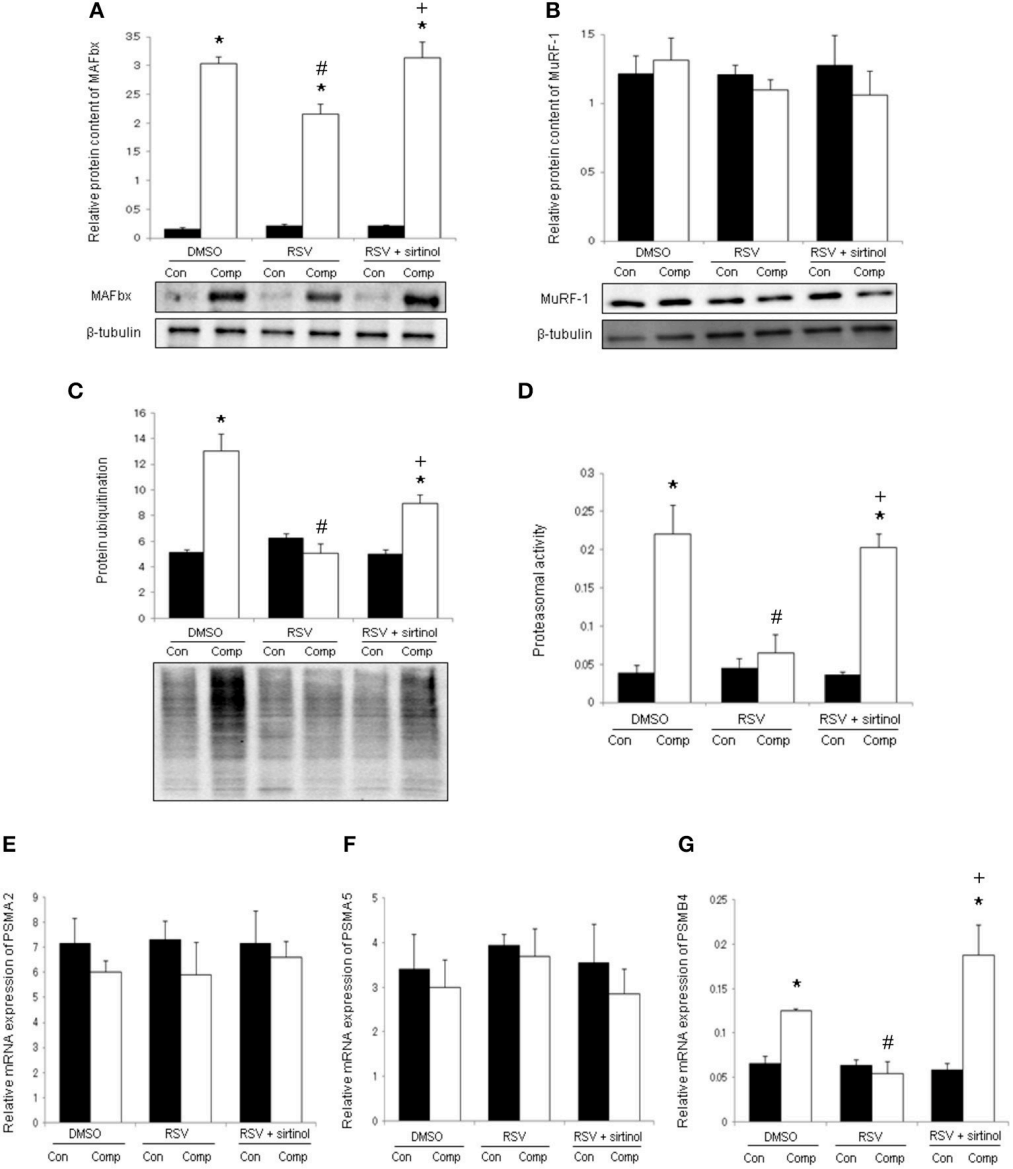
**Markers of muscle catabolism**. In the absence of SIRT1 inhibition, resveratrol down-regulated MAFbx in the compressed muscle **(A)**. The protein expression of MuRF-1 was not affected by compression or any of the treatments **(B)**. Resveratrol, but not concomitantly with sirtinol (5 mg kg^−1^), suppressed the compression-induced elevations of ubiquitinated proteins **(C)** and proteasomal activity **(D)**. The transcript levels of proteasomal alpha 2 subunit; PSMA2 **(E)**, alpha 5 subunit; PSMA5 **(F)** and beta 4 subunit; PSMB4 **(G)** were determined by qPCR. ^*^p < 0.05, compressed muscles (comp) compared to uncompressed control muscles (con); ^#^*p* < 0.05, resveratrol relative to DMSO; ^+^*p* < 0.05, resveratrol + sirtinol compared with resveratrol.

The protein content of MAFbx was elevated abruptly by 19.4 folds in the skeletal muscle in response to moderate compression (Figure 5A). This compression-induced increase was mitigated significantly by resveratrol only in the absence of SIRT1 inhibition (Figure 5A). However, the protein expression of MuRF-1 was not significantly different between control and compressed muscles of all treatment groups (Figure 5B). Protein ubiquitination was up-regulated by 2.5 folds in compressed muscles relative to uncompressed controls whereas this increase was reversed by resveratrol, but not in combination with sirtinol (Figure 5C). The proteasomal activity in compressed muscles was 5.8 folds higher relative to control muscles of DMSO group whereas this increase was abrogated by resveratrol, but not in conjunction with sirtinol treatment (Figure 5D). We also examined the alpha 2, alpha 5, and beta 4 subunits of proteasome at transcript level in which were denoted thereafter as PSMA2, PSMA5, and PSMB4, respectively. The mRNA levels of PSMA2 and PSMA5 were neither affected by compression nor any of the drugs studied (Figures 5E,F). The transcript content of PSMB4 was increased significantly by 91% in compressed muscles relative to uncompressed controls; this compression-induced increase was antagonized by resveratrol, but not in combination with sirtinol (Figure 5G).

## Discussion

This study has provided the novel insight that restoration of SIRT1 activity by resveratrol protected against the development of pathohistological damages in the skeletal muscle induced by moderate compression through the inactivation of apoptotic/catabolic pathways. Protein kinase B, also known as Akt1, is a well-accepted survival signal. In agreement with the observation that restoration of Akt phosphorylation in the skeletal muscle averted the productions of cleaved caspase 3 and 14-kDa actin fragment after angiotensin-II infusion (Song et al., 2005), resveratrol maintained the level of phosphorylated Akt and inhibited markers of apoptosis and muscle degradation in compressed muscles. The increase in interstitial space in response to compression could be a consequence of accumulation of tissue fluid that accompanied infiltration activities during inflammatory response. Earlier study has shown that the mRNA level of TNF-α, a member of inflammatory signaling was elevated with lesion size in a rat model of spinal cord injury (Lee et al., 2003). A former work from our laboratory has demonstrated consistently that the muscle contents of ED1 and ED2, which are macrophage markers, were elevated after compression (Teng et al., 2011). Recent research has exhibited that resveratrol blunted the level of TNF-α in the skeletal muscle of rats with chronic obstructive pulmonary disease (Qi et al., 2014); although whether the anti-inflammatory property of resveratrol would account for its modulatory effects on the reduction of interstitial space in compressed muscles shall be addressed in the future.

Elevations of apoptotic and catabolic signaling pathways have been implicated in various muscle pathologies. Forced-expression of Bcl2 was observed to ameliorate the loss of grip strength in a mouse model of oculopharyngeal muscular dystrophy (Davies and Rubinsztein, 2011). Supporting the inhibitory role of Bcl2 in Bax/Bak-mediated apoptosis (Dominov et al., 2005), the immunoreactivities of diffuse cytochrome c and active caspase 3 were concomitantly attenuated in dystrophic muscles expressing the Bcl2 transgene (Davies and Rubinsztein, 2011). The thought that anti-catabolic interventions would alleviate muscle injury was corroborated by a study showing that genetic inactivation of cellular inhibitor of apoptosis 1 (cIAP1), which is an ubiquitin ligase, reduced significantly the centronucleation of muscle fibers and infiltration of macrophages in soleus muscles of dystrophin-deficient mice (Enwere et al., 2013). Indeed, our laboratory has previously shown that markers related to apoptotic signaling and muscle degradation were elevated in the skeletal muscle of rats exposed to moderate compression (Siu et al., 2009, 2011). The present work is the first attempt to demonstrate that resveratrol, which is a natural polyphenol in grapes and red wine prevented the increases in protein abundances of p53, Bax, MAFbx, and ubiquitin and enzymatic activities of caspase 3 and proteasome induced by compression injury in the skeletal muscle. These results were in line with those reported with pharmacological inhibition of caspases (Teng et al., 2011) and proteasome (Siu et al., 2011). To our surprise, the increase in Bim protein after compression was not reversed by acute resveratrol treatment; this observation does not agree with our recent efforts showing that long-term resveratrol supplementation blunted the protein level of Bim in the senescent heart (Sin et al., 2014) and skeletal muscle (Sin et al., 2015b). While these discrepancies could be ascribed to the differences in the duration of resveratrol treatment, there are also evidences that resveratrol induced accumulation of Bim in the mitochondria and p53-independent cell death (Gogada et al., 2011); hence endeavors further efforts to dissect the regulatory mechanisms of Bim by resveratrol in muscle disorders.

It is thought that the pleiotropic actions of resveratrol are mediated largely by sirtuin 1 (SIRT1). Transcriptional repression of SIRT1 has been shown to diminish the anti-hypertrophic effects of resveratrol in cardiomyocytes stimulated with phenylephrine (Kuno et al., 2013). Previous studies also revealed that Nox4, which is a catalytic subunit of the oxidant-producing NADPH oxidase, was up-regulated in C2C12 cultures treated with SIRT1 siRNA (Hori et al., 2011). These data were in concordant with the findings exhibiting that resveratrol reduced the expression of acetyl-histone H3, a marker indicative of increased SIRT1 deacetylase activity; and abundances of nitrotyrosine and 8-OHdG in dystrophic muscles (Hori et al., 2011). While our previous efforts have shown that resveratrol ameliorated the compression-induced increases in nitrotyrosine and 4HNE in the skeletal muscle (Sin et al., 2013), here we further demonstrated that these resveratrol-induced suppressions were abolished by co-administration with sirtinol; thereby indicating a modulatory role of SIRT1 in the pathogenesis of deep tissue injury. Notably, our findings that the protein level of SIRT1 was reduced with concomitant up-regulation of MAFbx, which is an ubiquitin-related E3 ligase, in response to compression were consistent with those reported with food deprivation (Lee and Goldberg, 2013). The notion that SIRT1 inhibited muscle degradation was strengthened by the observation that forced expression of SIRT1, but not its deacetylation-defective mutant, abrogated the increase in Bnip3 transcript (Lee and Goldberg, 2013). It is also noteworthy that resveratrol enhanced the protein expression and deacetylase activity, but not the mRNA content of SIRT1 in compressed muscles. Despite that the reasons underlying this inconsistent mode of regulation are not understood, over-expression of miR-22 in the heart induced a remarkable decrease in SIRT1 protein whereas no overt changes at transcriptional level were detected (Gurha et al., 2013); it is therefore tempting to investigate whether inhibition of miR-22 would represent a novel myoprotective mechanism of resveratrol.

Foxo transcription factors are known to regulate the expression of apoptotic and atrophic genes. While earlier efforts have elucidated that the promoter binding activity of Foxo1 was compromised in Foxo1^AAA∕Arg215^ mutants (Schmoll et al., 2000) and that the transcript level of Bim was decreased in myotubes transfected with Foxo1^AAA∕Arg215^ (McLoughlin et al., 2009); the current work provided the first evidence that the increase in nuclear Foxo3a was paralleled by transactivation of Bim in the skeletal muscle during compression injury. We also found that resveratrol blunted the decreases in cytoplasmic levels of phosphorylated and total Foxo3a in the compressed muscle; which could be plausibly accounted by the degradation of Foxo3a following its deacetylation by SIRT1 and subsequent preferential binding of Skp2 subunit of E3 ubiquitin ligase (Wang et al., 2012). Another line of evidence has documented that a 48-h fasting induced functional deteriorations of the heart in Foxo1(3A/LXXAA) mice with impaired SIRT1-Foxo1 interaction but not in the wild type counterparts (Hariharan et al., 2010). Taken together, SIRT1-mediated deacetylation of Foxo1 may represent an important therapeutic mechanism of resveratrol in muscle disorders; although whether these findings would be extrapolated in compression-induced injury in the skeletal muscle warrant further research.

### Conflict of interest statement

The authors declare that the research was conducted in the absence of any commercial or financial relationships that could be construed as a potential conflict of interest.
